# Editorial comment – fungi-mediated bioremediation and bioconversion

**DOI:** 10.1080/21501203.2024.2370140

**Published:** 2024-07-09

**Authors:** Wensheng Qin, Sarita Shrestha

**Affiliations:** Department of Biology, Lakehead University, Thunder Bay, Canada

The population in the world is increasing continuously, and this increase directly or indirectly affects waste production, including agricultural waste. The agricultural waste obtained from various sources, such as farm waste and residues, forest waste, and municipal solid waste, is lignocellulose-rich biomass. The degradation and utilisation of lignocellulosic biomass by microorganisms have gained significant attention in recent years due to their potential to produce value-added bioproducts (Sadh et al. 2018; Shrestha et al. 2021). When the lignocellulosic biomass is not managed or disposed of properly, it may result in air, water, and soil pollution. In contrast, they are renewable and sustainable resources. They can be utilised to produce various value-added products to decrease pollution, reduce total production costs, and help in economic development and waste management (Ravindran et al. 2018). Biological methods for converting low-cost natural resources as economic substrates have been superior to other methods (physical and chemical methods) due to their availability, sustainability, and renewability (Saldarriaga-Hernández et al. 2020). Varieties of bacteria and fungi are being exploited in various research, including lignocellulolytic enzyme production (Saldarriaga-Hernández et al. 2020), remediation of heavy metal contaminated sediments (Dell’anno et al. 2022). However, recently, the exploitation of fungi in multiple studies has been an exciting subject for researchers and scientists. Different studies have demonstrated that fungi have the potential to convert biomass into a plethora of value-added products, such as antibiotics, bioactive compounds, biogas, etc., and could be even more effective than bacteria. A study illustrated that fungi overtook bacteria in the bioleaching of marine sediments contaminated highly with heavy metals and could be used in the bioremediation of contaminated heavy metals (Dell’anno et al. 2022). The bioconversion of lignocellulosic biomass by fungi not only provides an eco-friendly solution for waste management but also offers the possibility of producing valuable products such as biofuels, enzymes, and biopolymers (Shrestha et al. 2015). Additionally, the ability of fungi to degrade environmental pollutants such as pesticides, herbicides, and heavy metals makes them a promising candidate for environmental cleaning (Verma and Kuila 2019).

This special issue, therefore, aimed to include different topics such as:

- Bioremediation & Environmental Cleaning
- Fungi-Mediated Biocatalysis
- Lignocellulosic Biomass Bioconversion
- Microbial Biotechnology & Fermentation
- Value-added Bioproducts

In addition, the special issue tried to include, explore, and discuss the potential of fungi in diverse avenues in different original articles and a comprehensive review. It explored the various aspects of valorisation of lignocellulosic biomass using fungal species, including bioconversion of lignocellulosic biomass to produce value-added bioproducts, and their role in environmental cleaning (bioremediation of contaminated soils, water, and air). Huang et al. (2024) included fermentation technologies for enzyme production and strain modification of fungi, besides lignocellulosic enzyme production from agricultural wastes exploiting fungi. The included review article has described different agricultural wastes: food crop waste, cash crop waste, fruit waste, forestry waste, and various pretreatment methods (Huang et al. 2024). Furthermore, this issue demonstrated that eco-friendly sustainable biocomposites were produced using renewable and sustainable substrates like textile waste and spent coffee grounds. Those biocomposites can potentially replace polyester-based products and enhance packaging material’s properties (Kohphaisansombat et al. 2024; Saini et al. 2024). In addition, in this issue, Supmeeprom et al. (2024) used sawdust-based spent mushroom substrate, considered a lignocellulosic waste after mushroom production. They produced a sustainable value-added product called xylooligosaccharides using *Aspergillus flavus* KUB2 (Supmeeprom et al. 2024). The issue also highlighted key fungal species and various biochemical pathways for waste conversion processes (Dhiman et al. 2024). The article unravelled the lignocellulosic biomass-based circular economy concept, challenges and limitations, and critical components of the policy framework for efficient lignocellulosic biomass valorisation (Dhiman et al. 2024). Therefore, in a nutshell, this issue aids in expanding and understanding the role of varieties of fungi in valorisation and hopes to advance efficient and reliable technologies for economic development and waste management.
